# Bio-Hybrid Magnetic Robots: From Bioengineering to Targeted Therapy

**DOI:** 10.3390/bioengineering11040311

**Published:** 2024-03-26

**Authors:** Qian Zhang, Yun Zeng, Yang Zhao, Xuqi Peng, En Ren, Gang Liu

**Affiliations:** 1 Institute of Artificial Intelligence, Xiamen University, Xiamen 361005, China; 31520211154113@stu.xmu.edu.cn (Q.Z.); zenyun163@163.com (Y.Z.); zhaoy@xmu.edu.cn (Y.Z.); gangliu.cmitm@xmu.edu.cn (G.L.); 2 State Key Laboratory of Vaccines for Infectious Diseases, Center for Molecular Imaging and Translational Medicine, Xiang An Biomedicine Laboratory, School of Public Health, Xiamen University, Xiamen 361005, China; 3 Key Laboratory of Advanced Drug Delivery Systems, Zhejiang Province College of Pharmaceutical Sciences, Zhejiang University, Hangzhou 310058, China

**Keywords:** magnetic robotics, bioengineering, natural organisms, drug delivery, targeted therapy

## Abstract

Magnetic robots possess an innate ability to navigate through hard-to-reach cavities in the human body, making them promising tools for diagnosing and treating diseases minimally invasively. Despite significant advances, the development of robots with desirable locomotion and full biocompatibility under harsh physiological conditions remains challenging, which put forward new requirements for magnetic robots’ design and material synthesis. Compared to robots that are synthesized with inorganic materials, natural organisms like cells, bacteria or other microalgae exhibit ideal properties for in vivo applications, such as biocompatibility, deformability, auto-fluorescence, and self-propulsion, as well as easy for functional therapeutics engineering. In the process, these organisms can provide autonomous propulsion in biological fluids or external magnetic fields, while retaining their functionalities with integrating artificial robots, thus aiding targeted therapeutic delivery. This kind of robotics is named bio-hybrid magnetic robotics, and in this mini-review, recent progress including their design, engineering and potential for therapeutics delivery will be discussed. Additionally, the historical context and prominent examples will be introduced, and the complexities, potential pitfalls, and opportunities associated with bio-hybrid magnetic robotics will be discussed.

## 1. Introduction

Micro- and nanorobotics have gained attention for their potential in minimally invasive disease diagnosis and treatment due to their precise navigation capabilities in complex biological fluids [1,2,3,4]. However, the miniaturization process poses engineering challenges such as power sourcing, actuation control, multifunctional integration, and post-injection recovery or biodegradation capabilities. Researchers have employed various strategies to address these challenges, including the use of external fields (magnetic [5], ultrasound [6], and light [7]), biological motors [8], and chemical fuel [9] for actuation and motion control. Magnetic actuation, in particular, has garnered significant attention for its wireless power transmission ability, enabling high penetration through the human body without harm, even at relatively high field strengths [10,11,12].

To leverage the advantages of magnetic actuation, researchers have designed diverse magnetic formulations that are suitable for external magnetic fields and biomedical applications. These magnetic robots can be designed into arbitrary shapes, such as microspheres, ellipsoids, helices, and filaments [13]. Guided by external magnetic fields, they can navigate through complex geometries filled with different biological media, including water, blood, serum, mucus, urine, and gastric acid [14]. While these magnetic formulations demonstrate sensibility and practicability, they need to meet requirements such as traceability, biodegradability, and bioactivity to be versatile tools for in vivo applications. Therefore, there is a need to explore new materials with efficient autonomous motion ability, controllability, extended life span, and negligible toxicity for the synthesis of robots.

In response to these requirements, researchers are exploring natural living organisms as engines for the development of bio-hybrid magnetic robots [15]. Magnetotactic bacteria, for instance, possess inherent magnetic materials that can be utilized for drug delivery with the aid of an external magnetic field [16]. By incorporating synthetic superparamagnetic iron oxide nanoparticles (SPION) into other natural organisms, these robots can be powered by cellular actuation or external magnetic fields [17,18]. This capability allows them to generate strong, enduring propulsion for self-movement at high velocities, and to detect and react to changes or stimuli in their environment, replicating biological behaviors and showcasing advanced functionalities. Furthermore, various techniques for functional modifications have been devised to impart unique traits to these bio-hybrid magnetic robots, such as magnetic maneuverability, efficient drug loading, controlled release, and adjustable interaction with the targeted cells [19,20,21,22]. These advancements position bio-hybrid magnetic robots as highly promising tools for disease diagnosis and therapy.

**Figure 1:**
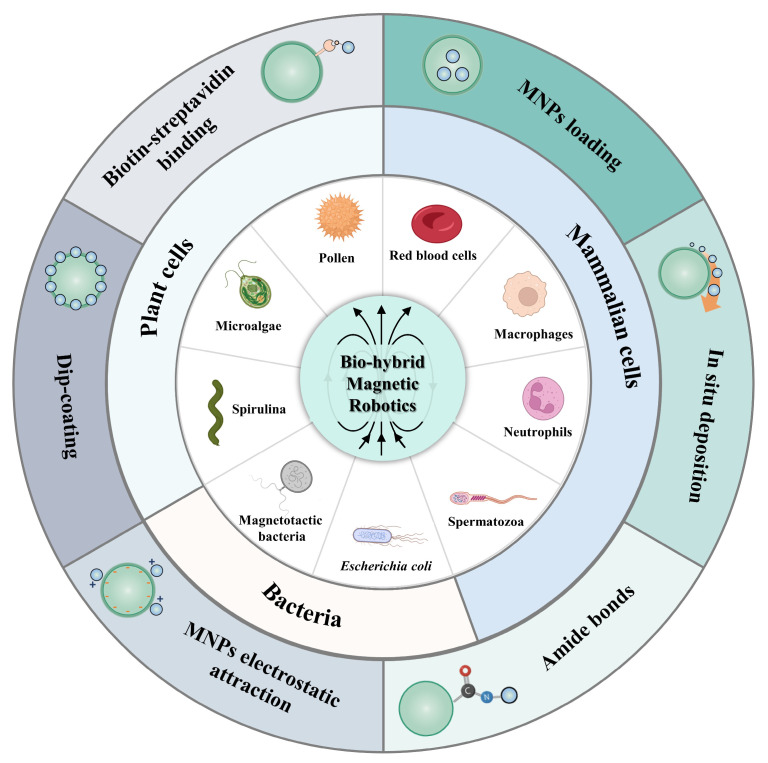
Schematic illustration of prepared bio-hybrid magnetic robots for targeted therapy. Compared with robots that are synthesized with inorganic materials, natural organisms like cells, bacteria, or other microalgae exhibit ideal properties for in vivo applications, such as biocompatibility, deformability, auto-fluorescence, and self-propulsion, as well as ease for functional therapeutics engineering.

This review focuses on discussing the design, fabrication, functionalization, motion capabilities, and performance of bio-hybrid magnetic robots for targeted delivery (Figure 1). According to the living organisms used, the bio-hybrid robots are categorized into three main groups: mammalian cells, plant cells, and bacteria. And prominent examples within each category will be introduced, highlighting their potential in targeted drug delivery. Also, several magnetic decoration methods for bio-hybrid magnetic microrobots’ synthesis will be summarized, such as magnetic nanoparticle (MNP) loading, in situ deposition, amide bonds modification, MNP electrostatic attraction, and dip-coating, as well as biotin-streptavidin binding. Furthermore, the review will address the complexities, potential pitfalls, and opportunities associated with bio-hybrid magnetic robots in this field, providing insights for future research and development.

## 2. Mammalian Cells

In recent years, there has been a growing emphasis on developing biomimetic micro/nanorobots using mammalian cells, due to their superior biocompatibility and safety compared to bacteria and plant sources [23,24]. This has spurred increased efforts in incorporating natural cell components into artificial miniaturized devices, such as utilizing cell membranes derived from living cells to functionalize micro/nanorobots or directly employing living cells to engulf them [25]. These approaches empower micro/nanorobots to adopt the surface membrane structures, biological substances, and functions of the source cells, while retaining their synthetic capabilities like propulsion through external fields and light-triggered drug release [26]. Moreover, these advancements have opened up avenues for precise cancer therapy and immune modulation by leveraging cell types such as red blood cells, neutrophils, macrophages, and sperm cells. In this context, strategies for engineering cellular nanorobots to overcome complex biological barriers and immune suppression will be discussed, along with their applications in immunotherapy, sonodynamic therapy, chemotherapy, and phototherapy.

### 2.1. Red Blood Cells

Among cell-based carriers, red blood cells (RBCs) are particularly noteworthy due to their abundant supply, unique mechanical properties, surface immunosuppressive characteristics, and versatile drug-carrying capacity [27,28,29]. Since the initial study in 1973, RBCs have been extensively researched and utilized as carriers, leveraging their immunosuppressive properties and multifunctional capacity to transport drugs within their structure [30,31]. Red blood cell (RBC) motors, incorporating SPION, demonstrate remarkable stability while preserving the native cell functionalities [5]. Despite their advantageous properties, the micron-scale dimensions of RBCs pose challenges in their ability to diffuse beyond blood vessels and interact directly with tumor cells in the context of imaging or treating solid tumors. This limitation hinders the achievement of tumor-cell-specific drug delivery through ligand–receptor interactions. However, the application of magnetic carriers can address these limitations by utilizing an externally applied magnetic field to enhance the accumulation of therapeutic agents within the tumor site, offering a promising solution [32]. Moreover, these RBC motors, which maintain the biological and structural characteristics of regular RBCs, exhibit a wide range of antigenic, transport, and mechanical properties that surpass those of typical synthetic motors [33,34].

**Figure 2:**
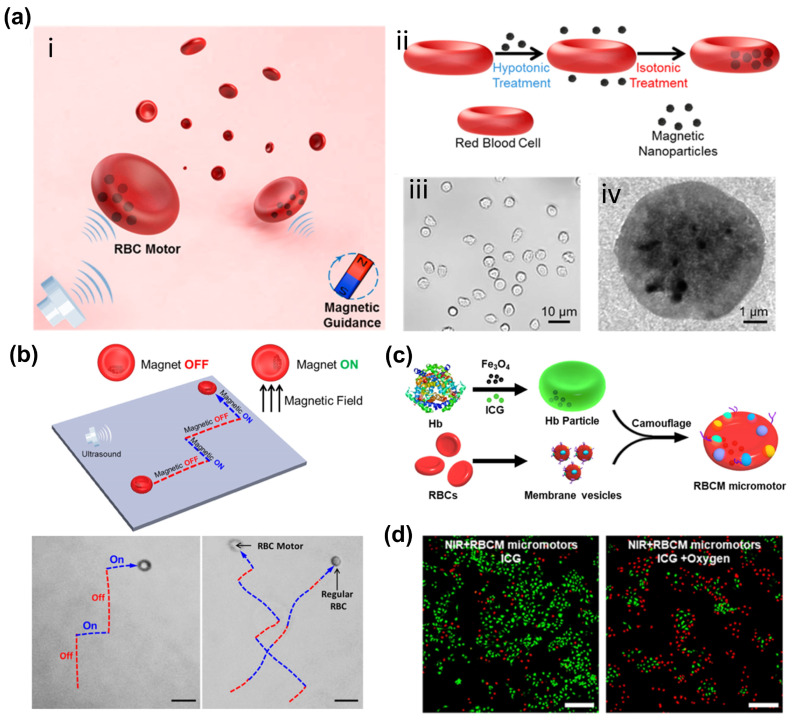
The preparation of red blood cells (RBCs) based bio-hybrid magnetic robots and their applications in drug delivery. (**a**) Schematic illustration of RBC-robots. (**i**) Magnetically guided and ultrasound-propelled RBC-robots. (**ii**) Preparation of the RBC-robots. (**iii**) Optical and (**iv**) TEM images of the synthesized RBC-robots. Magnetic nanoparticles loaded-RBCs showed well cellular structure. (**b**) Magneto-switchable guidance of ultrasound-powered RBC microrobots (Scale bar, 20 μm) [33]. Copyright 2014 American Chemical Society. (**c**) Schematic preparation of red blood cell-mimicking robots. (**d**) Cell viability staining images of HeLa cells after different treatments (Scale bar, 250 μm) [37]. Copyright 2019 American Chemical Society.

Additionally, Guo et al. (2020) conducted a study demonstrating that RBC-based robots retained essential features of natural RBCs, such as their oxygen-carrying capacity, size, shape, deformability, and prolonged circulation time, highlighting their potential for effective biomedical applications (Figure 2a,b) [35]. Also, the development of multifunctional RBC-based robots, loaded with quantum dots (QDs), the anti-cancer drug doxorubicin (DOX), and SPION, present a significant advancement in theranostic technology [36]. This innovation allows for the simultaneous treatment and monitoring of diseases, showcasing the versatility and potential impact of these microrobots in clinical settings. By leveraging RBC-based microrobots to encapsulate hemoglobin and deliver oxygen to tumor tissues, researchers have demonstrated enhanced efficacy in photodynamic therapy (PDT) for treating cancers (Figure 2c,d) [37]. The integration of RBC-based microrobots with various therapeutic agents and imaging probes establishes a flexible platform that holds promise for both disease treatment and diagnosis.

In addition to the significant progress in utilizing cells as carriers for drug delivery, cellular membrane vesicles offer distinct advantages over their parent cells in drug delivery. These advantages include the native cavity structure and size for drug loading, diverse modification tactics for the membrane surface, and naturally negligible immunogenicity in blood circulation. Building on these advantages, RBC membrane coating technology has been employed in the development of biomimetic bio-hybrid micro/nanorobots [38]. Researchers have documented the creation of a RBCs membrane-encased nanowire by combining biocompatible gold nanowire motors with RBC derived membrane vesicles, demonstrating effective acoustical propulsion. Furthermore, the use of an external magnetic field to induce targeted aggregation of magnetic RBCs based on physical force presents a highly feasible solution for enhancing the accumulation of therapeutic factors at tumor sites [39]. Additionally, through the assembly of negatively charged SPION into membrane-cloaked micro-robots via electrostatic attraction, researchers have been able to easily control the movement directions and paths of these microrobots. Moreover, the attachment of folate ligands to the surface of RBC vesicles via FR interaction has been demonstrated to enhance the efficacy of tumor magnetic targeting and facilitate the delivery of targeted drugs to ovarian cancer cells [40]. These advancements highlight the potential of leveraging cellular membrane vesicles in drug delivery, and underscore the promising role of RBC membrane coating technology in advancing targeted drug delivery systems. The multifaceted applications of this technology showcase its potential to revolutionize targeted drug delivery, particularly in the treatment of cancer and other diseases.

### 2.2. Neutrophils

Neutrophils are capable of migrating to sites of inflammation during the early stages of an immune response and responding rapidly to diseases within an inflammatory environment [41,42,43]. This has led to significant exploration of engineered neutrophils as a universal delivery vehicle for combating oncogenic diseases [44]. For instance, Shao et al. have successfully created a self-guided bio-hybrid robots by harnessing the inherent chemotactic capabilities of natural neutrophils, enabling autonomous navigation along a chemoattractant gradient for targeted drug delivery purposes [45]. To achieve targeted drug delivery, it is crucial to navigate biological barriers and reach sites of inflammation or infection. In another example, Zhang and colleagues have developed dual-responsive hybrid nanoelectromechanical (NE) robots capable of traversing the blood–brain barrier, allowing direct delivery of therapeutic agents to malignant glioma sites [46]. By exerting controllable intravascular movement in response to a rotating magnetic field, neutrophil-based robots demonstrate autonomous aggregation within the brain and subsequent traversal of the blood–brain barrier, which employ positive chemotactic motion along the gradient of inflammatory factors. These examples highlight the potential of utilizing engineered neutrophils as a promising delivery vehicle for combating various diseases. The utilization of neutrophils in bio-hybrid magnetic robotics provides a mean for targeted drug delivery, and their inherent chemotactic capabilities enable efficient navigation towards sites of inflammation or infection [47]. Moreover, this kind of bio-hybrid magnetic robot, which can maneuver through biological barriers such as the blood–brain barrier presents a promising approach for delivering therapeutic agents directly to malignant gliomas.

It is the same with the RBCs; researchers also use leukocyte membranes as a coating material to synthesize the bio-hybrid magnetic robots, which provides several advantages due to their ability to prolong the circulation duration of robots in biological fluids, effectively recognize cancer cells, and accumulate at tumor sites [48,49]. This not only offers a sophisticated platform for cancer diagnosis, but also presents promising prospects for therapeutic interventions. Unlike the “ultra-stealth” biomimetic coating provided by RBCs, neutrophils play a crucial role in resolving inflammation and repairing tissue damage through their unique actions [50]. Zhang et al. developed magnetosomes coated with a leukocyte membrane pre-engineered with azide (-N_3_) [51]. Upon intravenous injection, the membrane camouflage not only enables prolonged circulation, but also facilitates the loading of hydrophobic TGF-β inhibitor onto the membrane and conjugation of dibenzocyclooctyne (DBCO)-modified PD-1 antibody via a mild and efficient click chemistry process, creating an immunogenic tumor microenvironment (TME). This innovative strategy capitalizes on the multi-functionality of leukocyte membranes, allowing for enhanced targeting and delivery of therapeutic agents specifically to tumor sites. Additionally, the ability to modulate the TME using engineered leukocyte membrane coatings opens up new avenues for immunotherapeutic approaches against cancer.

Overall, the utilization of leukocyte and its membranes as coating materials presents a promising approach for targeted drug delivery and immunotherapeutic interventions against cancer. The multi-functionality of these hybrid magnetic robots allows for enhanced recognition and accumulation at tumor sites, while their ability to modulate the TME provides a means to improve the efficacy of therapeutic interventions.

### 2.3. Macrophages

**Figure 3:**
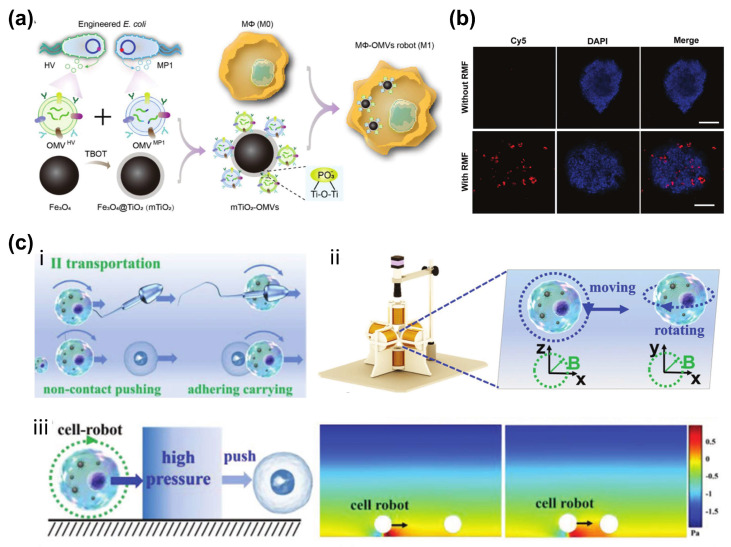
Schematic illustration of preparing macrophage based bio-hybrid magnetic robots and their applications in targeted therapy. (**a**) Schematic of the fabrication of M1 type robots. Two types of genetically engineered bacterial outer membrane vesicles (OMVs) were captured by functional MNPs, which were then endocytosed by macrophages to create M1−OMVs robots. (**b**) Fluorescence images depict the binding of Cy5−labeled cell robots to DAPI-labeled 3D tumor cell spheroids, both in the absence and presence of RMF control (Scale bars: 50 µm) [53]. Copyright 2023 John Wiley and Sons. (**c**) (**i**) Manipulation of various loads using a cell robot through both noncontact and contact methods. (**ii**) Schematic of the magnetic generating system which controls the movement and rotation of robots. (**iii**) Analysis and simulation of the noncontact transportation [54]. Copyright 2022 John Wiley and Sons.

Macrophages, another type of immune cell, possess superior phagocytic capabilities compared to other cell types, making them ideal candidates for loading SPION while maintaining their viability [52]. A noteworthy example of this is the work conducted by Li et al., who developed biogenic macrophage-based robots that are capable of targeted and multimodal cancer therapy through magnetic manipulation (Figure 3a,b) [53]. This innovative approach involves the utilization of magnetic nanoparticles and bioengineered bacterial outer membrane vesicles (OMVs). The macrophage cell robots exhibit remarkable control flexibility, even in complex environments, showcasing their potential as versatile and precise therapeutic agents. Additionally, these macrophage cell robots have demonstrated not only the ability to transport natural cells, but also their efficacy in noncontact transportation of mouse sperm (Figure 3c) [54]. This remarkable adaptability and functionality highlight the promising role of macrophage cell robots in various biomedical applications beyond cancer therapy.

It is different from the aforementioned neutrophils and RBCs; macrophages have the ability to assume distinct phenotypes, known as M1 and M2, depending on the specific microenvironment they encounter [55]. The M1 phenotype is associated with the inhibition of cancer cell growth, while the M2 phenotype promotes it [56,57]. Differences in their functions lead directly to differences in the protein composition of their cell membranes. To harness this phenotypic variability, researchers have developed a novel approach using M1 macrophage membrane-coated magnetic photo-thermal complexes (MPN) for tumor therapy guided by photoacoustic (PA) imaging [58]. By leveraging both the inherent properties of macrophages and the immune characteristics of M1 macrophage cells, the MPN demonstrates exceptional potential as a targeted therapy for tumors. This impressive fusion of macrophage membrane coating and magnetic photo-thermal properties not only enables specific tumor targeting, but also enhances the therapeutic efficacy, making them promising tools for effective anticancer treatment.

### 2.4. Sperm

Nature has ingeniously evolved flagella over millions of years to serve as delivery systems [59]. Among the various microscopic cells that employ this propulsion mechanism, sperm cells are particularly remarkable for their speed and efficiency. In recent years, the integration of synthetic micro- and nanomaterials with spermatozoa has given rise to tubular and helical sperm robotics, holding great promise for applications in the biomedical and engineering fields, such as assisting artificial insemination and drug or gene delivery [60]. Typically, a human sperm cell exhibits a beating frequency of 10–30 Hz, but this can vary depending on environmental conditions. Other swimming characteristics of human sperm include a velocity of approximately 50 µm/s, a beat amplitude of about 5 µm, and a beat wavelength of 12 µm [61,62]. Bovine sperm cells have been used as an exemplar of mammalian spermatozoa in initial demonstrations of sperm robotics due to their similar morphology to human sperm cells [63]. It is intriguing that spermatozoa possess the ability to flexibly alter their shape, enabling them to navigate smoothly through narrow and curved passages. Given their exceptional capability to maneuver within viscous microenvironments, they are highly suitable for propelling micro-robots [64].

**Figure 4:**
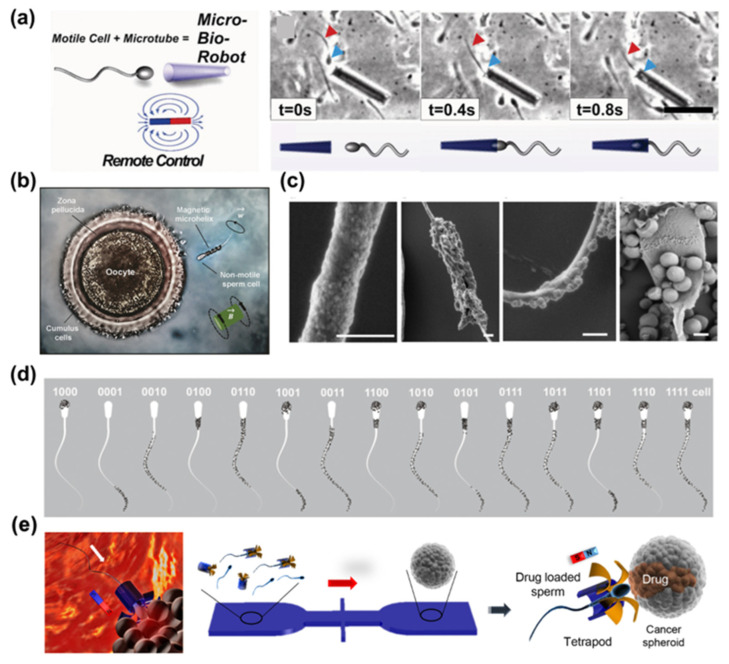
Several classical strategies to prepare bio-hybrid magnetic robots with sperm. (**a**) Diagram of the coupling process with a bull spermatozoon inside a magnetic microtube. The blue arrows point at sperm head, red arrows at flagella [65]. Copyright 2013 John Wiley and Sons. (**b**) Schematic of a captured immotile sperm delivered to the oocyte for fertilization [66]. Copyright 2016 American Chemical Society. (**c**) Cryo-SEM images depict positively charged SPION adhering to the surface of bovine spermatozoa (Scale bars: 1 µm) [68]. Copyright 2019 John Wiley and Sons. (**d**) Overview of potential configurations of sperm-templated robots based on the attachment of SPION to different segments of a sperm cell [70]. Copyright 2021 John Wiley and Sons. (**e**) Schematic illustration of the assist of microfluidic chip in drug-loaded sperm transport and delivery [71]. Copyright 2018 American Chemical Society.

In 2013, the study of sperm-based magnetic robotics began with the use of a 50 µm-long rolled-up ferromagnetic microtube to precisely guide single spermatozoa to predetermined positions (Figure 4a) [65]. Microtubes are large enough to prevent cellular uptake, allowing for their combination with sperm cells without affecting their activity or ability to undergo acrosome reactions. Similarly, single spermatozoa can be captured and guided using helical microstructures fabricated by direct laser writing (Figure 4b) [66]. Another proposed method to develop bio-hybrid robots involves using spiral microstructures as biological templates through a dip-coating process in magnetite suspensions [67]. Iron oxide micro/nanoparticles can easily adhere to the surface of sperm cells through electrostatic self-assembly (Figure 4c) [68]. In a previous study, an artificial robotic sperm was developed through electrospinning, resulting in a flexible polystyrene tail with a magnetic head [69]. This millimeter-sized robotic sperm was activated using oscillating magnetic fields, and demonstrated a forward motion of up to 0.9 body lengths per second. However, it should be noted that the charge distribution on the sperm membrane is non-uniform (Figure 4d) [70]. Despite the overall negative net charge of the sperm cell, distinct regions of positive charge, primarily located on the sperm heads, exist. The cells are completely coated with 1 mm iron oxide particles, resulting in limited bending amplitude along the flagellum [68]. Smaller iron oxide particles are unevenly distributed along the head, mid-piece, principal piece, and distal end of the sperm cell. Consequently, the flexibility of the flagellum is maintained to facilitate the propagation of flagellar waves.

Furthermore, sperm robots have demonstrated their ability to deliver anticancer drugs locally to cancer spheroids. Robots have also been utilized to carry or guide sperm cells for assisted in vivo fertilization (Figure 4e) [71]. Despite being incapacitated or dead, sperm’s structural properties remain advantageous for drug delivery purposes. Misra and co-workers introduced hybrid robots that utilize sperm cells for targeted drug delivery [72]. The system comprises a motile sperm cell as the propulsion source and drug carrier, along with a 3D-printed magnetic tubular microstructure known as a “tetrapod”. With controlled guidance and release mechanisms, these robots show promise in delivering drugs specifically to tumor cells while minimizing accumulation in healthy tissues. Notable features include high drug loading capacity, self-propulsion capability, and enhanced drug availability. However, loading magnetic nanoparticles in sperm, although fabricated from a sperm template and propelled at frequencies comparable to live cells, results in robots with a maximum swimming speed six times lower than that of natural sperms. Overall, sperm robotics demonstrates the potential of flexible, bio-templated magnetic micro-swimmers for application in minimally invasive procedures within the human body.

## 3. Plant Cells

In contrast to mammalian cells, plant cells possess distinctive features such as a rigid cell wall and chloroplasts. The cell wall serves to maintain the structural integrity of the cell, while chloroplasts enable the capture of sunlight and conversion of it into chemical energy through photosynthesis. Due to their explicit structure and ease of cultivation, plant cells have been increasingly utilized in the synthesis of magnetic robots. In this section, we will discuss two representative examples: microalgae and pollen.

### 3.1. Microalgae

**Figure 5:**
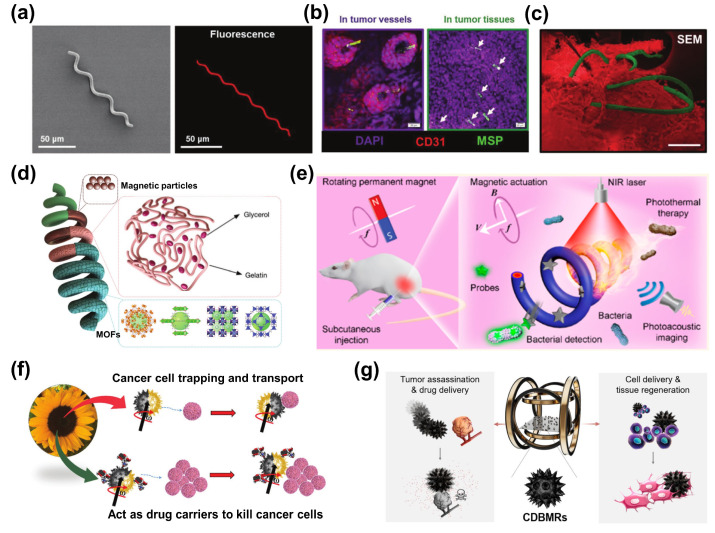
Diagram of preparing bio-hybrid magnetic robots with microalgae and pollen, as well as their applications in targeted therapy. (**a**) SEM images (left) and fluorescence (right) images of *S. platensis.* (**b**) The stained tumor tissues post i.v. injection of magnetic *S. platensis*. Tumor tissues stained with DAPI (purple, nuclei) and CD31 (red, vessels). Scale bar: 20 µm. (**c**) SEM image of tumor tissue (pseudocolored red) and the magnetic *S. platensis* (pseudocolored green) (Scale bar: 100 µm). The write arrow points the MSP in tumor tissue [78]. Copyright 2020 John Wiley and Sons. (**d**) Illustration of MOF-based microrobot [80]. Copyright 2021 John Wiley and Sons (**e**) Schematic illustration of a polydopamine (PDA)-coated magnetic microswimmer consisting of a magnetized *Spirulina* (MSP) matrix and PDA surface for the treatment of bacterial infection [81]. Copyright 2020 American Chemical Society. (**f**) Schematic representation of magnetic sunflower pollen-based biological robots [89]. Copyright 2022 John Wiley and Sons. (**g**) Schematic illustration of bio-hybrid magnetic microrobots (CDBMRs) for active drug delivery, active cell delivery, and tissue regeneration [90]. Copyright 2022 American Chemical Society.

Microalgae are a group of photosynthetic organisms that are mainly present in aquatic environments, and contain various photosynthetic pigments, such as chlorophyll, which allows them to harness sunlight and convert it into chemical energy through photosynthesis [73,74]. Green algae-based bio-hybrid robots have numerous advantages over synthetic robots, including simple production processes, sustained propulsion without external actuation, and easy cargo conjugation [75]. In addition, the microalgae exhibit diverse structural shapes that aid in generating motion in a specific magnetic field environment. For example, spirulina contains natural helical structures that can convert rotational motion into translational motion in fluid, which is particularly advantageous in overcoming the influence of high viscosity in biological media on robots’ motion [76,77]. Furthermore, spirulina’s uniform helical structure provides robust propulsion, natural fluorescence, tailored biodegradation, and selective cytotoxicity, making it an efficient and accurate model for developing bio-hybrid robots (Figure 5a) [78]. Additionally, spirulina’s negatively charged surface can attract molecules and particles with opposite charges, making it a versatile carrier for synthesizing various delivery systems. As a magnetized microswimmer, magnetic *S. platensis* could navigate through tumor vasculature and accumulate in tumor tissues (Figure 5b,c).

To exploit the characteristics of microalgae for the synthesis of robots, Zhang’s group reported a bio-hybrid magnetic robot (BMR) made from spirulina platensis (*S. platensis*) in 2017 [79]. The BMR demonstrated intrinsic fluorescence, MR signals, natural degradability, and desirable cytotoxicity. Subsequently, multifunctional spirulina-based robots have been developed for disease imaging and therapy. For example, a photosynthetic bio-hybrid nanoswimmer system has the potential to serve as an oxygenerator for in situ O_2_ generation in hypoxic solid tumors, thereby modulating the tumor microenvironment (TME) and enhancing the efficacy of radiotherapy [78]. Furthermore, metal–organic framework (MOF) nanocrystals can assemble into gelatin magnetite-coated microalgae, and gelatin’s thermal responsiveness aids in releasing magnetic bio-templates from the MOF nanocrystal cargoes, facilitating targeted drug delivery in cancer cells (Figure 5d) [80]. The properties of existing robots are further enhanced by the introduction of a poly-dopamine coating, leading to improved photoacoustic (PA) signals and photo-thermal effects, enabling PA image tracking and photo-thermal therapy (Figure 5e) [81]. As a proof of concept, real-time image tracking through PA imaging and the desired theranostic capabilities of microswimmer swarms are showcased for the treatment of pathogenic bacterial infections. The development of spirulina-based robots offers promising opportunities in the fields of drug delivery and disease therapy.

### 3.2. Pollen

In addition to the whole cells for the synthesis of bio-hybrid magnetic robots, other components derived from the plants are also used, and pollen is the most representative example. Pollen serves as a natural micro-carrier for the transfer of cellular content within plants’ reproductive organs [82,83,84]. With a wide range of shapes and sizes, pollen grains of the same species exhibit characteristic features, including uniformity in size, dispersity, and shape. In recent years, pollen has gained significant attention as a drug delivery system due to its simplicity, low cost, large surface-to-volume ratio, high loading capacity, and high bioavailability [85]. The diverse array of pollen across plant species allows for the selection of suitable pollen with the desired shape and size for various applications [86].

The concept of pollen-based robotics was initially introduced by Pumera’s group, resulting in a versatile platform for drug delivery and water purification [87]. They successfully developed nine distinct pollen robots derived from natural pollen grains, which served as carriers for cargo storage and delivery [88]. Additionally, these kinds of bio-hybrid magnetic robots were created by depositing a ferromagnetic metal layer on one side of the pollen surface (Figure 5f) [89]. Surprisingly, the researchers discovered that these magnetic sunflower pollen-based bio-hybrid magnetic robots possessed an inherent ability to attract cancer cells due to opposite charges. Consequently, pollen-based bio-hybrid magnetic robots exhibited the capability to attract, manipulate, and eliminate ovarian cancer cells using a transversal rotating magnetic field. Similarly, Liu et al. designed chrysanthemum pollen-derived bio-hybrid magnetic robots with spiny protrusions, hollow cavities, and porous surface structures for tumor eradication and active tissue regeneration (Figure 5g) [90]. Through a sequential treatment process, the microstructure displayed an enlarged hollow cavity, providing sufficient space for cargo encapsulation. Furthermore, drug-loaded bio-hybrid magnetic robots could be magnetically guided to tumor tissue and anchored onto cell surfaces using burr-like micro-spikes for adhesion. Subsequently, a time-varying magnetic field can induce internal cell destruction and release loaded drugs through high-frequency vibration.

Apart from tumor eradication, pollen-based bio-hybrid magnetic robots hold significant potential for treating bacterial biofilms on medical implants. Sun et al. developed magnetic urchin-like capsule robots loaded with magnetic liquid metal droplets, resulting in synergistic biofilm eradication without the development of drug resistance [91]. The feasibility and efficacy of these microswarms in eliminating bacterial biofilms in clinical biliary stents were demonstrated. It is worth noting that pollen is generally recognized as safe material by the Food and Drug Administration (FDA), making it more easily approved for use in oral and gastrointestinal pharmaceutical preparations [92]. With its favorable biocompatibility, biodegradability, and controlled release behavior, pollen-based magnetic robots offer an innovative approach to effective disease treatment and hold great potential for clinical applications.

## 4. Bacteria

Various bacteria possess innate propulsion and sensing abilities that enable taxis-based self-guidance, making them ideal for intelligent bio-hybrid robotics [93,94]. By integrating functional nano-hybrids, bacteria-based robots can serve as smart drug delivery carriers, responding to varied simulation signals such as chemotaxis or magnetotaxis to reach target regions. In recent years, significant progress has been made in the fabrication, propulsion, imaging, and therapy of bacteria-based bio-hybrid magnetic robots for various diseases [95,96]. Two types of magnetically responsive bacteria are commonly used: those rendered magnetic through conjugation with magnetic materials, and magnetotactic bacteria (MTB) with innate magnetic properties. Notably, bacteria have long been identified in many human cancer types, and their ability to home in on hypoxic areas of solid tumors presents unique advantages for targeted therapy. Bacteria can directly carry chemotherapeutic drugs or integrate drug-carrying vehicles to enhance antitumor efficacy [97]. Genetic modifications can also be made to express therapeutic genes and reporter genes for tumor therapy and in vivo imaging. Bacteria like *Escherichia coli*, which possess multiple peritrichous flagella, can be bio-engineered into microrobots for noninvasive targeted delivery in physiological environments. This section summarizes the advancements in two main types of bacteria-based robots (*Escherichia coli* and MTB) and their biomedical applications.

### 4.1. Magnetotactic Bacteria

**Figure 6:**
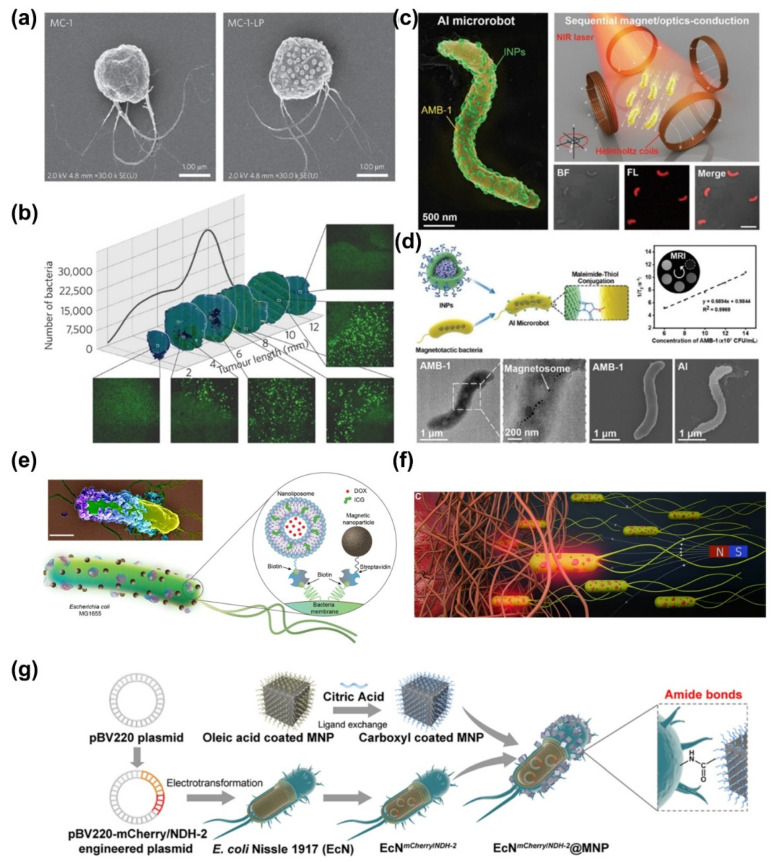
Schematic preparation of bacteria-based bio-hybrid magnetic robots and their applications in targeted therapy. (**a**) The TEM image of MC-1 and MC-1 decorated with liposome, in which the drugs can be loaded. (**b**) Transverse tumor sections of MC-1 with liposome anchoring after targeting. Images of each section were acquired using a fluorescence optical microscope, and images show a good distribution of the loaded MC-1 cells throughout the tumor [100]. Copyright 2016 Springer Nature. (**c**) SEM image of a representative AMB-1 based microrobots and the schematic of this kind of microrobots in sequential conduction under magnetic/optical fields. Scale bar: 5 µm. (**d**) Preparation and characterization of AMB-1 based robots with sequential magneto/optics-conducted capability. The robots were fabricated with the AMB-1 facilely coated with the photosensitizer via a Michael addition reaction [101]. Copyright 2021 John Wiley and Sons. (**e**) Schematic illustration of the *E. coli*-based bio-hybrid robots that are conjugated with nickel nanoparticles (NLs) and SPION. These NLs are loaded with doxorubicin (DOX) and indocyanine green (ICG). (**f**) Conceptual schematics illustrating bacterial bio-hybrid robots being magnetically guided through porous microenvironments towards specific target tissues, such as tumors [105]. Copyright 2022 Akolpoglu, M.B some rights reserved. (**g**) Schematic illustration for the preparation of engineered *E. coli*-based hybrid magnetically robots with triple-perception capabilities for the magnetic, thermal, and hypoxic environments [106]. Copyright 2022 American Chemical Society.

Magnetotactic bacteria (MTB) have the remarkable ability to biomineralize stable magnetized anisotropic chains of magnetite nanocrystals, derived from their natural aquatic habitats [96]. These bacteria employ magnetically assisted aerotaxis as a means of navigating towards regions characterized by low oxygen levels [98]. Considering the inherent resistance of hypoxic tumor regions, where oxygen depletion hampers the effectiveness of conventional therapies, a promising approach involves the utilization of modified targeting agents capable of directed migration towards these hypoxic areas for the purpose of delivering therapeutic payloads [99]. In a groundbreaking advancement, Sylvain Martel and colleagues have harnessed magnetically guided nanorobotic agents, based on *Magnetococcus marinus* MC-1 cells, to facilitate the transportation of drug-loaded nano-liposomes into hypoxic tumor regions (Figure 6a,b) [100]. These nanorobots exhibit magneto-aerotactic migration behavior and possess dimensions suitable for deep tumor penetration under the influence of a directed magnetic field. Experimental findings have demonstrated that when MC-1 cells carrying drug-loaded liposomes were magnetically guided in proximity to tumors in animal models, up to 55% of the cells successfully reached hypoxic regions within colorectal xenografts. Furthermore, each MC-1 cell was found to carry approximately 70 drug-loaded liposomes, highlighting the potential of these bio-hybrid magnetic robots to enhance therapeutic efficacy within tumor hypoxic regions.

In addition to MC-1, the *Magnetospirillum magneticum* AMB-1 has also been employed in the fabrication of bio-hybrid magnetic robots (Figure 6c,d) [101]. By modifying the surface of AMB-1 bacteria, liposomes encapsulating indocyanine green (ICG) were attached. The AMB-1 bacteria possess the ability to autonomously swim towards tumor sites through internal hypoxia-driven effects in conjunction with an externally applied magnetic field. Simultaneously, ICG serves as a fluorescence imaging agent and a photo-thermal therapy agent. This specific design enables the magnetic robot to track its movement using fluorescence and magnetic resonance imaging (MRI). Experimental studies have demonstrated that this magnetic robot can sequentially migrate towards the hypoxic internal regions of tumors and effectively eliminate solid tumors through photo-thermal therapy under laser irradiation. Notably, the AMB-1 surface can be decorated with dopamine and synthetic Fe_3_O_4_ magnetic nanoparticles, allowing for the production of semi-artificial magnetotactic bacteria [102]. Through this approach, the magnetic properties of these bacteria, including saturation magnetization and residual magnetization, can be regulated in a multivariate manner, resulting in alterations in magnetic sensitivity. This strategy provides a feasible method to maneuver MTB for applications in complex fluid environments, such as magnetic drug release systems and real-time tracking systems.

### 4.2. Escherichia coli

Bacteria-driven bio-hybrids are a notable category of robots due to their efficient flagellar propulsion, ability to navigate within intricate bodily tissues, and sensing capabilities of physiological and pathophysiological gradients. Notably, *Escherichia coli* Nissle 1917 (EcN) bacteria, compared to other bacteria employed in the construction of hypoxia-targeted bacteria micro-robots, are nonpathogenic Gram-negative probiotics [103]. These bacteria possess a unique ability to specifically target and identify signal molecules in the hypoxic regions of tumors. Consequently, EcN has been modified into a self-propelled robot to surmount biological or pathological barriers for the purpose of delivering therapeutic payloads [104]. However, many fabrication techniques utilized in the design of bio-hybrid robots have detrimental effects that can disrupt the intrinsic properties of bacteria, including swimming speed, taxis, and membrane protein expressions.

A study by Metin Sitti demonstrated the construction of bacterial bio-hybrids through harmless, noncovalent interactions, which enabled the attachment of synthetic materials such as streptavidin magnetic materials, nanoparticles, and biotinylated nano-liposomes to motile bacteria (Figure 6e,f) [105]. Remarkably, the addition of these artificial units did not compromise the original swimming velocity of the bacterial bio-hybrid robots. To address the challenges in collective perception and precise propulsion in fluids, Cheng’s group reported an Escherichia coli-based bio-hybrid robot with triple-perception capabilities for the magnetic, thermal, and hypoxic environments, thereby enhancing tumor-targeting ability (Figure 6g) [106]. The experimental feedback, based on fluorescent protein imaging, indicated that the robot exhibited strong thermal sensitivity and active targeting prowess towards the tumor area in a collective manner under the influence of a magnetic field.

Furthermore, advancements in genetic engineering tools for bacteria have provided unprecedented opportunities for enhancing the capabilities of biological units, including reduced cytotoxicity, targeted surface molecule expression, collective remote perception, and precise propulsion. A rapid, specific, and efficient labeling approach for micro-particle attachment was reported in the fabrication and application of bacteria based robots [107]. Specifically, the engineered auto-transporter antigen 43 was introduced into *Escherichia coli* to display biotin on its surface, utilizing bacterial motility to significantly expedite the attachment of streptavidin-coated cargo particles. Additionally, microbial surface display technologies can be employed to exhibit other proteins for diverse functions. For instance, the Mms6 protein is displayed on the bacterial surface to facilitate magnetite bio-mineralization [108]. This approach presents a flexible and customizable strategy for constructing bacteria-based robots, offering a reliable and versatile solution for their design.

## 5. Conclusions and Prospects

The field of medical microrobotics traces its origins back to Richard Feynman’s concept of “swallowing a surgeon” [109]. Despite numerous advancements in medical technology, the goal of achieving noninvasive disease cures through robots remains largely aspirational. Over the past decade, significant progress has been made in utilizing magnetic actuation for medical applications [110,111,112]. However, there are still considerable challenges to overcome in the design, fabrication, sensing, and control of magnetic robots. In contrast to synthetic materials, bio-hybrid magnetic robots based on natural bioactive materials offer several key advantages. Firstly, natural selection has endowed real-life organisms with intricate biological materials that surpass current technological capabilities. Microalgae, bacteria, and cells exhibit unique structures and diverse functionalities, including hydrophobic/hydrophilic properties, biodegradability, magnetism, phototropism, lightweight characteristics, and self-fluorescence [113]. Secondly, bio-hybrid magnetic robots demonstrate exceptional biodegradability, which is crucial for biomedical applications [114]. The persistence of non-degradable robots in the body can lead to undesirable cytotoxic effects and necessitate invasive removal methods like surgery, causing harm to the organism [115]. These attributes make bio-hybrid magnetic robots promising for targeted therapeutics in vivo, garnering increased attention and research interest.

Although the field of leveraging bio-hybrid magnetic robots for biomedical applications, such as cancer treatment and drug delivery, has been on the rise, there are still limitations to current technology. The ideal magnetic robots should possess high biocompatibility, ease of manufacturing, controllable drug release, precise manipulation, high loading capacity, and promising clinical prospects. Designing robots as all-in-one integrated solutions that can be customized to meet individual patient needs is crucial. However, several crucial issues need to be addressed to ensure technological advancement, including therapeutic efficacy, magnetic manipulation, biosafety, and clinical translation. The efficient loading of drugs into biological materials and the precise targeting of drug release are significant hurdles to overcome [116,117]. Magnetic actuation and manipulation platforms play a vital role in magnetic controllable applications [118,119,120]. Biosafety is essential to ensure the safety and effectiveness of bio-hybrid magnetic robots, and several factors such as genotoxicity, immuno-toxicity, and neurotoxicity require further research [121,122]. Finally, the cost-effectiveness of these robots for medical applications and the feasibility of mass production need further evaluation, along with government policy support for approval as new drugs or medical devices [123,124].

This review consolidates information on bio-hybrid magnetic robots based on natural materials, and outlines current progress in their biomedical applications. Just as summarized in Table 1, the basic parameter for various microrobots has been highlighted. The natural materials primarily include animal cells, plant cells, bacteria, and fungi. The advantages and disadvantages of bio-hybrid magnetic robots as active drug delivery carriers are discussed, along with the main challenges in therapeutic efficiency, maneuverability, biosafety, and clinical translational feasibility. While acknowledging the substantial challenges in developing bio-hybrid magnetic robotic systems and their limited current clinical potential, there is optimism for their future application in precision medicine to enhance patient diagnosis and treatment. Collaboration among researchers from academia and industry, spanning diverse expertise across materials science, chemistry, physics, mechanics, robotics, and biomedicine, will be crucial due to the interdisciplinary nature of this emerging field. The future focus will involve creating automated platforms that integrate multiple functionalities within complex biological systems, forming closed-loop systems by combining highly integrated micro/nanorobotics with suitable magnetic actuation and manipulation platforms. Anticipated progress in these areas holds the promise of significant advancements in related fields.

## Figures and Tables

**Table 1:** A summary of major applications and functionalities of bio-hybrid magnetic robots.

Bio-Hybrid Magnetic Robots	Size	Drugs	Driven Force	Magnetic Decoration	Application	Velocity	Ref.
Rebuilt red blood cells (RRBCs)	~20 μm	Mn-TPPS4, DOX	Permanent magnet	MNPs loading	MRI contrast imaging, therapeutic drug delivery	-	[35]
RBC-based micromotors	~6–8 μm	Quantum dots/doxorubicin	Ultrasound	MNPs loading	Therapeutic and diagnostic agents	15 ± 2 μm/s	[36]
Magnetically navigated red blood cell-mimicking (RBCM) micromotor	2 μm	Photosensitizers	Ultrasound	MNPs loading	Photodynamic cancer therapy	56.5 μm/s	[37]
RBC@magnetic mesoporous silica nanoparticles (MMSNs)	~100 nm	Hypocrellin B	Permanent magnet	MNPs loading	Cancer therapy	-	[39]
DOX-loaded glucose/gluconic acid-coated magnetic nanoparticles	91.2 ± 20.8 nm	Doxorubicin/glucose/gluconic acid	Permanent magnet	MNPs loading	Cancer therapy	-	[40]
Dual-responsive biohybrid neutrobots	~105 nm	Paclitaxel	Electromagnetic system	MNPs loading	Active target delivery	16.4 μm/s	[46]
Engineered magnetosomes	~100 nm	PD-1 antibody/TGF- β inhibitor	Permanent magnet	No	Cancer therapy	-	[51]
Magnetic-propelled macrophage-based microrobots	~90 nm	-	Electromagnetic system	MNPs loading	Cancer therapy	25.9 μm/s	[53]
Macrophage template-based microrobots	15–20 µm	-	Electromagnetic system	MNPs loading	Object transportation	-	[54]
M1 macrophage membrane-camouflaged magnetic nanorobots	182 ± 3 nm	Doxorubicin/black phosphorus quantum dots	Electromagnetic system	MNPs loading	Cancer chemo-phototherapy	10 μm/s	[58]
Micro-bio-robot	50 μm × 5–8 μm	-	Permanent magnet	Loading	Micromanipulation	10 μm/s	[65]
Sperm-Templated Microrobots	~65 μm	-	ATP driven force	Electrostatic attraction	-	15.6 ± 3.6 μm/s	[70]
Sperm-hybrid micromotor	10 μm	Doxorubicin	ATP driven force	Loading	Targeted drug delivery	41 ± 10 μm/s	[71]
IRONSperm	~60 μm	Doxorubicin	Electromagnetic system	Electrostatic attraction	Targeted drug delivery	6.8 ± 4.1 µm/s	[72]
Biohybrid nanoswimmers system (PBNs)	~150 μm × 5 μm	-	Permanent magnet	Dip-coating	Radio-photodynamic therapy	78.3 μm/s	[78]
Biohybrid magnetic robot (BMR)	~150 μm × 5 μm	-	Electromagnetic system	Dip-coating	Fluorescence and MR imaging-guided therapy	90 μm/s	[79]
MOF-based microrobot (MOFBOT)	-	Doxorubicin	Electromagnetic system	Dip-coating	photocatalytic degradation	-	[80]
Magnetic microswimmers	~100 μm × 5 μm	-	Electromagnetic system	Dip-coating	Antibacterial therapy	-	[81]
Sunflower pollen-based BioBot (SFPµP-BioBots)	~25 μm	Doxorubicin	Rotating magnetic field	Electrostatic attraction	Cancer therapy	24.9 μm/s	[89]
Chrysanthemum pollen-derived biohybrid magnetic microrobots (CDBMRs)	~30 μm	Doxorubicin	Rotating magnetic field	In situ deposition	Active drug delivery	-	[90]
Magnetic urchin-like capsule robots (MUCRs)	~25 μm	L-aspartic acid	Electromagnetic system	Loading	Biofilm eradication	2 mm/s	[91]
Magnetococcus marinus strain MC-1	1–2 μm	Drug-containing nanoliposomes	Electromagnetic system	No	Active drug delivery	-	[100]
Magnetospirillum magneticum (AMB-1)	~2 μm × 0.5 μm	Indocyanine green nanoparticles	Electromagnetic system	No	Targeted cancer therapy	13.3 ± 4.5 μm/s	[101]
Semiartificial magnetotactic bacteria (SAMTB)	~3 μm × 0.5 μm	-	Electromagnetic system	In situ deposition	Cargo-transportation	-	[102]
Bacterial biohybrid microrobots	-	Doxorubicin and indocyanine green	Electromagnetic system	Biotin-streptavidin binding	Stimuli-responsive cargo delivery	18.5 μm/s	[105]
Biohybrid microrobot	~5 μm	-	Rotating magnetic field	Amide bonds	Imaging-guided cancer treatment	25 μm/s	[106]
